# Victims’ use of professional services in a Dutch sexual assault centre

**DOI:** 10.3402/ejpt.v5.23645

**Published:** 2014-06-18

**Authors:** Iva Bicanic, Hanneke Snetselaar, Ad De Jongh, Elise Van de Putte

**Affiliations:** 1 National Psychotrauma Center for Children and Youth, University Medical Center Utrecht, Utrecht, the Netherlands; 2 Department of Behavioral Sciences, Academic Centre for Dentistry in Amsterdam (ACTA), University of Amsterdam and VU University in Amsterdam, Amsterdam, the Netherlands; 3 School of Health Sciences, Salford University, Manchester, United Kingdom; 4 Department of Paediatrics, University Medical Center Utrecht, Utrecht, the Netherlands

**Keywords:** Assault, sexual assault, emergency care, mental health, post-traumatic stress disorder

## Abstract

**Background:**

Prior research endorsed the establishment of sexual assault centres in the Netherlands because of the potential benefit for victims’ mental recovery. In 2012, the first Dutch sexual assault centre was founded at the University Medical Center Utrecht. The aim of the centre is to provide 24/7 coordinated and integrated services (i.e., medical, forensic, and psychological) in one location.

**Objective:**

The purpose of the present study was to describe demographic, background, and assault characteristics of victims seen at the centre within one week post-assault, and their use of post-assault services in order to improve current services.

**Method:**

From January 2012 to September 2013, prospective data of 108 patients were collected. To describe the population included, frequency counts and proportions were generated for categorical variables.

**Results:**

The mean age was 21.3 years (*SD*=9.8). Most victims were female (91.7%). A large proportion of victims reported background characteristics known to increase the risk for post-traumatic stress disorder (PTSD) and revictimisation such as prior sexual abuse (32.4%), pre-existing use of mental health services (45.4%), and not living with both biological parents (61.7%). Most patients (88.9%) consulted the centre within 72 hours post-assault. The uptake of services was high: 82.4% received emergency medical care, 61.7% underwent a forensic–medical exam, 34% reported to the police, and 82.4% utilised psychological services.

**Conclusion:**

To prevent revictimisation and PTSD, current psychological services could be improved with immediate trauma-focused treatments. Current forensic services may be improved with the use of standard top to toe forensic–medical examinations for both children and adults.

The experience of rape or sexual assault is affecting a considerable percentage of individuals in the Netherlands; 2.6% of men and 11.7% of women between 15 and 70 years reported a history of at least one rape in their lifetime (De Haas, Van Berlo, Bakker, & Vanwesenbeeck, 2012). Yearly, 0.4% of men and 2.3% of women older than 15 years are confronted with one or more sexual offences (Merens, Hartgers, & Van den Brakel, 2012). For women, 19% of these offences concern an attempted or completed rape.

Despite the potential negative impact of sexual assault on mental, sexual, and physical health (Linden, 2011; Postma, Bicanic, Van der Vaart, & Laan, 2013; Rees et al., 2011), and the high risk for revictimisation after assault (Littleton, Axsom, & Grills-Taquechel, 2009), evidence suggests that most victims do not use professional services and do not report the assault to the police (Campbell & Wasco, 2005; Wolitzky-Taylor et al., 2011). The only previous study that investigated post-assault services in the Netherlands demonstrated that victims recovered relatively slowly and they were confronted with post-rape services in various locations with waiting lists (Ensink & Van Berlo, 1999). However, those victims who were provided with immediate and integrated services reported improved mental recovery. Based upon these findings, Ensink and Van Berlo (1999) recommended the establishment of multidisciplinary sexual assault centres in the Netherlands like those existing in the United States and Scandinavian countries (Bramsen, Elklit, & Nielsen, 2009; Campbell, Patterson, & Lichty, 2005). More recent evidence confirms our experiences in clinical practice that coordinated assistance is more effective than a non-integrated approach in facilitating recovery from assault and increasing chances of the offender's apprehension (Campbell, Patterson, Adams, Diegel, & Coats, 2008; Campbell, Patterson, & Bybee, 2012). As a result, initiatives were taken to set up integrated services for acute sexual assault victims.

In 2012, the first sexual assault centre in the Netherlands was founded. This centre is located at the Emergency Department of the University Medical Center Utrecht (UMCU) and offers 24/7 medical, forensic, and psychological services to anyone who believes he or she has been the victim of a recent (i.e., <1 week) sexual assault. The available medical services aim to provide emergency medical care and subsequent follow-ups. The purpose of forensic services is to optimally perform the forensic examination within the time limits for evidence collection. The psychological services aim at reducing initial distress by means of “watchful waiting” (National Institute for Clinical Excellence [NICE], 2005) as the intensity of acute psychological reactions may play a role in later recovery, with higher levels of immediate distress associated with poorer outcome (Alisic, Jongmans, Van Wesel, & Kleber, 2011; Bryant & Panasetis, 2001). Watchful waiting is the recommended approach after a traumatic event as set out by the NICE guidelines, implying close monitoring of the patient without active treatment. In the case of young victims, parents or caregivers are provided with parallel psychological services, based on evidence suggesting that high levels of parental stress predict post-traumatic stress disorder in children (PTSD; Alisic et al., 2011).

To date, a total of four centres in the Netherlands apply the above multidisciplinary approach in the management of acute assault, suggesting that awareness of this approach is growing among clinicians and policy makers (Goderie & Flikweert, 2012; Vanoni, Kriek, & Linneman, 2013). However, a good understanding of the characteristics of acute assault victims and their use of services is necessary to improve the current services. Therefore, the present study uses the patient database of the UMCU sexual assault centre to describe victims’ demographic, background, and assault characteristics and their use of post-assault professional services (i.e., medical, forensic, and psychological services). The time-period to evaluate the use of post-assault services was 2 months, based on the time needed for PTSD treatment and for medical care including follow-ups.

## Methods

### Subjects and data collection

This study was conducted at the sexual assault centre in the UMCU, which focuses on providing multidisciplinary post-assault services within one week post-assault. All patients older than 16 years (and their parents or formal caregivers in case of children aged 0–16 years) who visited the centre between January 2012 and September 2013, and gave informed consent, were included in the present study. All information was anonymised, whilst specific details of the events were omitted.

In the sexual assault centre, patients are first triaged by a trained (forensic) nurse. Only patients who intend to report to the police go through a forensic–medical examination using an evidence sampling kit. Emergency medical care includes physical examination, documentation of injuries, provision of emergency contraception, and prophylactic treatment for sexually transmitted diseases (STDs) including HIV and hepatitis B and C. The (forensic) nurse informs the patient about medical follow-up services, such as HIV post-exposure prophylaxis (PEP) monitoring and side effect management, and testing of STDs, HIV, hepatitis B and C. One day after the visit, a case-manager is appointed to all consenting patients to coordinate follow-up appointments and to contact pre-existing mental health services. The case-manager is a mental health professional who is also responsible for the provision of psychological care according to the “watchful waiting protocol” (NICE, 2005) in the first month following assault. If necessary, evidence-based treatment for PTSD is provided such as cognitive behaviour therapy (CBT) or eye movement desensitisation reprocessing (EMDR) therapy. If the victim is a child, parents or caregivers are offered parallel psychological support, but children at the age of 16 years or older can consent to services at the centre without their parents being notified. In the UMCU, professionals involved in medical and psychological services use the same patient file.

Between January 2012 and September 2013, the sexual assault centre received phone calls and emails from police (15%), (mental) health services (52.5%), or self-referrals (32.5%) concerning 659 (alleged) assault victims. Their mean age was 17.7 years (*SD*=10.1). Most victims were female (86.8%). Of the phone calls and emails concerning 659 cases, 407 cases were not admitted to the UMCU because of long travel distance, living abroad, victim's concerns about anonymity, and resistance towards involvement of professionals. Admission to the UMCU for medical and/or psychological care was realised for 252 of the 659 cases (38.4%). Of these 252 cases, “time since assault” was more than one week for 144 cases. Specifically for this study, we included only admissions to the sexual assault centre that were within one week post-assault. This concerned 108 cases.

### Measurements

Information from the patient (mental) health record generated at time of admission was encoded into the sexual assault centre's database for the purpose of the present study.

Patient characteristics included gender, age, ethnicity, current living situation, family structure, use of pre-existing mental health services, intellectual disabilities, current pregnancy, current use of prescribed medication, refugee status, prior sexual trauma, prior physical abuse, and whether they were under custody of Child Protection Services.

Assault characteristics included type of sexual assault, physical violence, victim's intake of alcohol and/or drugs prior to assault, assailant's gender, (estimated) assailants’ age category, number of assailants, and relationship to the assailant.

Characteristics of post-assault services included prior use of the centre, number of services used (maximal 3), time since assault (in days), use of emergency medical care, involvement of a medical specialist, (treatment of) assault-related physical injury, prescription of medication, diagnostics of STDs, involvement of police, use of forensic–medical examination, official report made to the police, use of case-management, application of PTSD treatment, and put under custody of Child Protection Services.

### Data analyses

To describe the population included, frequency counts and proportions were generated for categorical variables. Statistical analyses were conducted using SPSS (IBM SPSS Statistics for Windows, Version 20.0. Armonk, NY: IBM Corp.).

## Results

Referral sources for the sample of 108 victims who were admitted to the centre within one week post-assault, included police (61.9%), (mental) health services (27.6%), and victims themselves (11.5%).

### Patient characteristics

The demographic and background characteristics of the 108 patients are presented in Table 1. Age ranged from 3 to 59 years, with a mean age of 21.3 years (SD =9.8). The age group between 12 and 25 years accounted for 68.5% of the sample. A substantial number of the sample reported prior sexual abuse (32.4%), prior physical abuse (29.6%), and pre-existing mental health services (45.4%). One-quarter (25.3%) was homeless or living in residential or foster care. Of the minors, 61.7% was not living with both biological parents.

**Table 1:** Demographic and background characteristics of the victims (*N*=108) at time of admission in frequencies and percentages

	*N*	%
Gender		
Female	99	91.7
Male	9	8.3
Age category		
<12 years	7	6.5
12–17 years	40	37.0
18–25 years	34	31.5
>25 years	27	25.0
Ethnicity^a^		
Western	95	88.0
Non-Western	13	12.0
Living situation		
Living with parent(s)	45	41.6
Living with partner	10	9.2
Living alone	17	15.6
Residential or foster care	21	19.3
Homeless	6	6.0
Missing	9	8.3
Refugee status	5	4.6
Prior sexual abuse	35	32.4
Prior physical abuse	32	29.6
Pre-existing use of mental health services	49	45.4
Pre-existing use of prescribed medication	53	57.6
Intellectual disability identified	15	16.2
Family structure—only minors included (*n=47)*		
Complete	14	29.8
Incomplete due to divorce or death	29	61.7
Missing	4	8.5
Under custody of Child Protection Services	13	27.7

a Western was identified as the country of birth being Europa, United States, or Australia.

### Assault and assailant characteristics

Assault and assailant characteristics are presented in Table 2. Penetrating assault occurred in 93.5% of the cases. For 77 of the 108 victims (71.3%), the index trauma concerned a single-incident sexual assault. For seven of the 108 victims (6.5%), the trauma concerned two incidents of sexual assault. For 20 of the 108 victims (18.5%), the trauma concerned a series of three or more incidents of sexual assault. Data about assault frequency were not available for four victims (3.7%). Most victims (86.1%) were assaulted by male adults. Group rape occurred in 24.1% of the cases. The majority of victims (83.3%) knew their assailant, who was most often a peer or a person close to them.

**Table 2:** Assault and assailant characteristics reported by victims (*N*=108) at time of admission in frequencies and percentages

	*N*	%
Type of assault		
Vaginal	53	49.1
Oral	7	6.5
Anal	4	3.7
Multiple	37	34.2
No penetration	7	6.5
Presence of physical violence	32	29.6
Victim's use of alcohol/drugs	24	22.2
Gender		
Female	1	0.9
Male	107	99.1
(Estimated) age category		
Minors (<18 years)	27	13.9
Adults	80	86.1
Number of assailants		
1	82	75.9
>1	26	24.1
Victim–assailant relationship		
Peer^a^	25	23.1
Intimate	19	17.6
Stranger^b^	18	16.7
Human trafficking	12	11.1
Acquaintance	12	11.1
Professional	10	9.3
Family	6	5.6
Prostitution	3	2.8
Internet contact	3	2.8

a Peer refers to a non-romantic relationship with a similar-aged person.

b Stranger in case the victim had never contacted the assailant before.

### Use of post-assault services

Post-assault service utilisation was categorised into three types: medical, police (including forensic–medical exam), and psychological. Ten patients (9.3%) used one out of three services, 29 (26.9%) used two out of three services, and 69 (63.9%) used all three services. The majority of patients (88.9%, *n*=96) presented at the centre within 72 hours post-assault, while 11.1% (*n*=12) presented at the centre 4–6 days post-assault. Details of post-assault service utilisation are presented in Table 3. Emergency medical care was utilised by 82.4% of the sample. All users of emergency medical care had experienced penetrating assault. Physical injury was reported by 23.1% (*n*=6 genital; *n*=19 non-genital) of the patients, of which 32% (*n*=8) were in need of injury treatment. Of the 50 patients using PEP medication, 54% did not complete the treatment scheme because of: severe side effects (*n*=3); interaction with other prescribed medication (*n*=1); assailant being diagnosed as HIV-negative (*n*=2); patient's perceived stress (*n*=2); ignorance regarding the use of medication (*n*=1); patient's own indication of having low infection risk (*n*=1); and unknown reasons (*n*=17). After pregnancy testing, it appeared that out of all fertile girls and women, one adult woman was pregnant.

**Table 3:** Details of post-assault services utilisation (i.e., medical, police, and psychological) by victims (*N*=108) in frequencies and percentages

	*N*	%
Prior use of the sexual assault centre	4	3.7
Medical services		
Emergency medical care	89	82.4
Specialist involved (not mutually exclusive groups)		
Infectious disease specialist	69	63.8
Gynaecologist—only females included (*n=*99)	12	12.1
Paediatrician—only minors included (*n=*47)	36	76.6
Assessment of the presence of STDs (including hepatitis C)	82	75.9
Medical interventions administered		
Antibiotics	74	68.5
Hepatitis B immunisation	69	63.8
HIV post-exposure prophylaxis (PEP)	50	46.3
Emergency contraception—only females included (*n=*99)	36	36.4
Forensic services		
Police involvement	92	85.2
Forensic–medical examination	66	61.7
Official report to police	36	34.0
Psychological services		
Use of case-management	89	82.4
Receipt of PTSD treatment	42	38.9
Parallel psychological care for parents—only minors included (*n=*47)	34	72.3

For forensic services, 61.7% of the patients underwent a forensic–medical examination and 34% decided to officially report the assault to the police.

Case-management was provided to 82.4% of the cases. Relatively more minors and their parents (*n*=42) consentedto a case-manager than adults (*n*=47). Evidence-based treatment of PTSD according to the Dutch multidisciplinary guidelines for the treatment of anxiety disorders (NICE, 2005) was provided to 38.9% of the patients within 2 months post-assault. After the assault, four minors were put under custody of Child Protection Services.

## Discussion

The present findings showed that most victims presenting at a hospital-based sexual assault centre within one week post-assault were females between 12 and 25 years, who experienced penetrating assault by a known male adult. The victims reported high rates of prior victimisation, pre-existing use of mental health services, use of prescribed medication and poor living conditions (i.e., housing and parental support). Their usage of post-assault services was high and one-third decided to officially report to the police. Considering that the sample consisted of severe cases with various needs, the multidisciplinary approach used seemed appropriate.

Although the Dutch approach towards the management of acute sexual assault victims has only recently changed from non-integrated to coordinated and integrated, the characteristics of the study population yielded results consistent with research in sexual assault centres in Denmark and the United States (Avegno, Mills, & Mills, 2009; Brown, Du Mont, MacDonald, & Bainbridge, 2013; Ingemann-Hansen, Sabroe, Brink, Knudsen, & Charles, 2009). To this end, the present study confirms female gender and adolescence as being the greatest risk factors for sexual assault (De Haas et al., 2012), which may reflect the lifestyles and circumstances of young women or their socialising with males.

A large proportion of victims reported past victimisation and pre-existing use of mental health services. This outcome is in line with prior studies in acute assault victims (Brown et al., 2013; Campbell, Keegan, Cybulska, & Forster, 2007; Elwood et al., 2011). These characteristics, as well as the finding that most minors were not living with both biological parents, have been found to be associated with an enhanced risk of PTSD onset and revictimisation (Classen, Palesh, & Aggarwal, 2005; McLaughlin et al., 2013; Walsh et al., 2012). Thus, recognition of these risk factors and application of appropriate psychological care is essential to prevent revictimisation and facilitate mental recovery.

Sixty-four percent of the sample utilised all three (i.e., medical, forensic, psychological) services. This high percentage may reflect the needs associated with sequelae of exposure to sexual assault. The uptake of emergency medical care was high as pregnancy and STD prevention are the two key acute threats for victims of sexual assault. General physical injury was observed in only one-quarter of the cases. This percentage is lower than findings from prior studies (Riggs, Houry, Long, Markovchick, & Feldhaus, 2000). This may be explained by the relatively small number of stranger assault in the present study, which has been found to be associated with physical injury (Riggs et al., 2000). Several explanations may be applicable for the finding that genital injury was observed in only 6% of the sample, while 94% reported penetration. The use of a colposcope was not routinely used, possibly resulting in less well documented genital injuries. It could be that the study population consisted of persons with prior sexual experiences (Grossin et al., 2003), a finding in agreement with what is known about female physiology. On the other hand, low numbers of genital injury have been found in previous studies among children (Palusci, Cox, Shatz, & Schultze, 2006). Further, half of the patients using PEP medication did not complete the treatment scheme despite education on the use and side effects of PEP. Therefore, concerted efforts should be made to improve treatment compliance.

The finding that one-third of the victims officially reported to the police is higher than the national report percentages of 10% (Merens et al., 2012). This may be explained by the fact that the police was the centre's primary referral source, but it could also be argued that the coordination of services might have contributed to this higher percentage (Campbell et al., 2012). Close collaboration between different and trained disciplines has been found to prevent victim blaming (Campbell et al., 2012) and, consequently, empower victims in their decision to report the assault event to the police. Further research is needed to examine whether a multidisciplinary approach increases numbers of police reporting in the Netherlands.

This study was set up to gain information about victims’ characteristics and their use of services in order to improve current post-assault services. Although almost all patients made use of the available psychological services, it could be argued whether this was the appropriate care. Watchful waiting relies on the assumption that most victims following a traumatic experience will improve without treatment within a few weeks. This is not to be expected in this sample considering the finding that many patients showed increased risk factors for PTSD and revictimisation, such as prior abuse, not living with both biological parents, and pre-existing use of mental health services (Acierno, Resnick, Kilpatrick, Saunders, & Best, 1999; Elwood et al., 2011; Littleton et al., 2009; McLaughlin et al., 2013; Rees et al., 2011; Walsh et al., 2012). The latter factor suggests the presence of premorbid mental health problems. Moreover, PTSD (symptoms) has been found to mediate revictimisation (Risser, Hetzel-Riggin, Thomsen, & McCanne, 2006). Thus, current psychological care may be improved with immediate evidence-based treatments, e.g., CBT and EMDR therapy, focused on resolving victims’ traumatic memories. To date, several pilot studies of brief psychosocial interventions in adult victims of recent rape have been conducted. A study with female assault victims showed that rates of PTSD severity were equivalent at a 9-month follow-up after either early CBT or supportive counselling, although early CBT accelerated the recovery process (Foa, Zoellner, & Feeny, 2006). A video-based intervention providing psycho-education to rape victims immediately before a forensic rape exam has shown preliminary support in the prevention of post-rape psychopathology (Resnick et al., 2007). One recent study (Zoellner, Feeny, Eftekhari & Foa, 2011) showed evidence supporting the effect of a brief cognitive-behavioural intervention in the recovery after assault. Finally, an initial feasibility study of an EMDR treatment for patients presenting to an emergency department (ED) within hours of a rape showed a reduction in post-traumatic stress and sexual problems, which remained stable at 4 weeks and 6 months after the intervention (Tarquinio, Brennstuhl, Reichenbach, Rydberg, & Tarquinio, 2012). Despite the encouraging results of these studies, at present there is no clear evidence-based preventive intervention for young and adult victims of recent rape. However, the need for preventive interventions is supported by recent evidence suggesting that PTSD symptoms should be targeted after sexual assault, because changes in PTSD symptoms is likely to influence subsequent changes in other psychological symptoms (Nickerson et al., 2012). In the present study, 39% of both young and adult patients received either CBT or EMDR therapy, though not immediately. Longitudinal follow-up is necessary to reveal information about optimal treatment timing and mental recovery rates, not only for PTSD symptomatology but also with regard to depression, anxiety and sexual problems. Also, in future early treatment effect studies, attention should paid to the assessment of dissociation as a recent study showed that dissociation at the time of the first treatment session was associated with reduced response to an early exposure intervention (Price, Kearns, Houry, & Rothbaum, 2014). Also, a meta-analysis by Ozer, Best, Lipsey, and Weiss (2003) found dissociation to be the strongest predictor of later PTSD among other variables including prior trauma, prior psychological adjustment, family history of psychopathology, perceived life threat during the trauma, and post trauma social support. The present study is considered a first step towards more elaborate data collection by describing victims’ characteristics.

There are some study limitations that should be mentioned. First, we suspect that reports of prior trauma are likely to have been conservative because patients were asked about their backgrounds without having established a relationship with the involved professional. Second, the forensic–medical examination of children and adults was not performed in similar ways. For adult victims, the focus of the forensic–medical examination is determined by victims’ disclosure to the police about the event. Children were routinely assessed top to toe while adults were not, which may have resulted in less well documented injuries in adults (Ingemann-Hansen et al., 2009). Despite these limitations, this is the first study in the Netherlands that extensively described characteristics of victims, both minors and adults, and their use of post-assault services in a designated sexual assault centre.

In conclusion, young women consulting a sexual assault centre appeared to have background characteristics that put them at risk for the development of PTSD and revictimisation. Therefore, appropriate psychological care is essential to prevent revictimisation and facilitate mental recovery. Possibly, current psychological services may be improved by immediately targeting PTSD symptoms with trauma-focused treatments. Improvement in forensic services may be achieved by performing standard top to toe forensic–medical examinations for both children and adults. Future research should investigate the potential benefit of sexual assault centres for (mental) health and the judicial process.
